# Calsenilin, a Presenilin Interactor, Regulates RhoA Signaling and Neurite Outgrowth

**DOI:** 10.3390/ijms19041196

**Published:** 2018-04-13

**Authors:** Hee-Jun Kim, Won-Haeng Lee, Mo-Jong Kim, Sunmee Shin, Byungki Jang, Jae-Bong Park, Wilma Wasco, Joseph D. Buxbaum, Yong-Sun Kim, Eun-Kyoung Choi

**Affiliations:** 1 Ilsong Institute of Life Science, Hallym University, Anyang, Gyeonggi-do 14066, Korea; hijuni@hallym.ac.kr (H.-J.K.); lwh1942@hallym.ac.kr (W.-H.L.); hanbami0730@hallym.ac.kr (M.-J.K.); actionsm@hanmail.net (S.S.); jang@hallym.ac.kr (B.J.); yskim@hallym.ac.kr (Y.-S.K.); 2 Department of Biomedical Gerontology, Graduate School of Hallym University, Chuncheon, Gangwon-do 24252, Korea; 3 Department of Biochemistry, College of Medicine, Hallym University, Chuncheon, Gangwon-do 24252, Korea; jbpark@hallym.ac.kr; 4 Genetics and Aging Research Unit, Mass General Institute for Neurodegenerative Disease, Department of Neurology, Massachusetts General Hospital, Harvard Medical School, Charlestown, MA 02129, USA; wasco@helix.mgh.harvard.edu; 5 Department of Psychiatry, Icahn School of Medicine at Mount Sinai, New York, NY 10029, USA; admin.buxbaum@mssm.edu; 6 Department of Microbiology, College of Medicine, Hallym University, Chuncheon, Gangwon-do 24252, Korea

**Keywords:** calsenilin, RhoA, p190RhoGAP, neurite outgrowth

## Abstract

Calsenilin modulates A-type potassium channels, regulates presenilin-mediated γ-secretase activity, and represses prodynorphin and *c-fos* genes expression. RhoA is involved in various cellular functions including proliferation, differentiation, migration, transcription, and regulation of the actin cytoskeleton. Although recent studies demonstrate that calsenilin can directly interact with RhoA and that RhoA inactivation is essential for neuritogenesis, it is uncertain whether there is a link between calsenilin and RhoA-regulated neuritogenesis. Here, we investigated the role of calsenilin in RhoA-regulated neuritogenesis using in vitro and in vivo systems. We found that calsenilin induced RhoA inactivation, which accompanied RhoA phosphorylation and the reduced phosphorylation levels of LIM kinase (LIMK) and cofilin. Interestingly, PC12 cells overexpressing either full-length (FL) or the caspase 3-derived C-terminal fragment (CTF) of calsenilin significantly inactivated RhoA through its interaction with RhoA and p190 Rho GTPase-activating protein (p190RhoGAP). In addition, cells expressing FL and the CTF of calsenilin had increased neurite outgrowth compared to cells expressing the N-terminal fragment (NTF) of calsenilin or vector alone. Moreover, Tat-C3 and Y27632 treatment significantly increased the percentage of neurite-bearing cells, neurite length, and the number of neurites in cells. Finally, calsenilin deficiency in the brains of calsenilin-knockout mice significantly interfered with RhoA inactivation. These findings suggest that calsenilin contributes to neuritogenesis through RhoA inactivation.

## 1. Introduction

Calsenilin was first identified as a binding partner of presenilin (PS) that regulates the levels of PS fragments [1]. It has also been reported that calsenilin acts as a transcription factor known as downstream regulatory element antagonist modulator (DREAM) in the nucleus. Many transcription factors are regulated by intracellular Ca^2+^, and calsenilin is one of the proteins that acts as a location-dependent gene silencer. Although calsenilin is a diffuse cytoplasmic protein, it can be localized in the endoplasmic reticulum (ER), Golgi apparatus and/or plasma membranes, and translocated into the nucleus in the presence of increased intracellular Ca^2+^ [2]. Calsenilin is cleaved by caspase-3, an event that generates a variable N-terminal fragment (NTF) and a conserved C-terminal fragment (CTF), the latter of which contains four Ca^2+^-binding EF-hand domains [3], and this cleavage is inhibited by the phosphorylation at serine residue 63 [4].

In transgenic mice, the functional loss of calsenilin has been implicated in abnormalities of synaptic plasticity, behavioral learning, and contextual fear memory, phenomena driven by the Ca^2+^-dependent negative regulation of the cyclic adenosine monophosphate (cAMP) response element-binding protein (CREB) and CREB-dependent transcription [5,6]. In addition, the absence of calsenilin expression slows the decline of neurons and memory function in aging [5]. However, the regulatory mechanism of calsenilin in synaptic plasticity and neuronal development remains unknown.

It has been reported that calsenilin can interact with Rho GTPases including Rabs, Rap1, and RhoA in a Ca^2+^-independent manner [7]. RhoA, a small GTPase protein, is involved in various cellular functions, including actin dynamics, cell proliferation, differentiation, migration, transcriptional regulation, cell cycle progression, and membrane trafficking [8,9,10,11], The activity of RhoA is regulated by a molecular switch cycling between the inactive GDP-bound (RhoA-GDP) and the active GTP-bound state (RhoA-GTP). RhoA is activated by guanine nucleotide exchange factors (GEFs; catalyze the exchange of GDP for GTP). However, GTPase-activating proteins (GAPs; stimulate GTPase activity), and guanine nucleotide dissociation inhibitors (GDIs; inhibit the exchange of GDP for GTP in cytoplasm) induce the inactivation of the proteins by enhancing the hydrolysis of the GTP-bound form and interfering with the exchange of GDP for GTP, respectively [12]. The GTP-bound and GDP-bound states of RhoA are controlled to maintain cellular homeostasis. RhoA and its regulatory proteins, namely p190RhoGAP and Rho GDP-dissociation inhibitor (RhoGDI), play an important role in neuronal differentiation, neurite outgrowth, neuronal migration, axon growth and dendritic spine formation and maintenance [13]. Previous studies have demonstrated that RhoA inactivation is essential for neuronal development and differentiation [14,15].

In this study, we investigated the role of calsenilin in RhoA-regulated neuritogenesis using in vitro and in vivo systems. We demonstrated that the overexpression of calsenilin induced neurite outgrowth by RhoA inactivation, through facilitating interaction of calsenilin with RhoA and/or RhoA regulatory proteins.

## 2. Results

### 2.1. Calsenilin Interacts with RhoA

**Figure 1:**
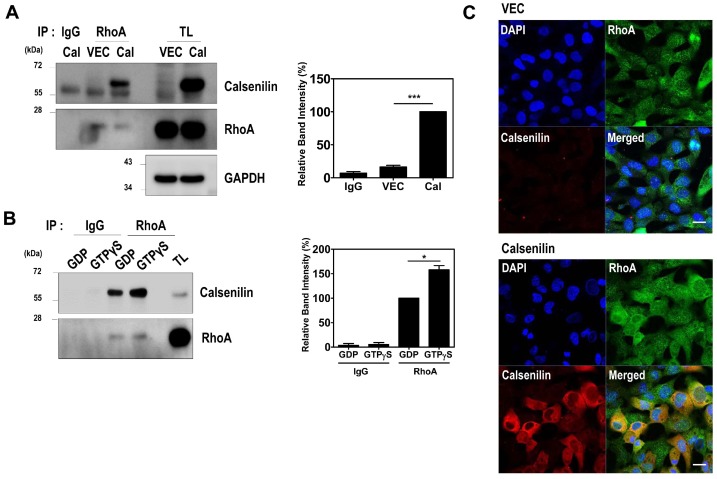
Calsenilin interacts with RhoA. (**A**) PC12 cells stably expressing either vector (VEC) or calsenilin (Cal) were immunoprecipitated with an anti-RhoA antibody and then analyzed by Western blotting with anti-calsenilin, and anti-RhoA antibodies; (**B**) Total cell lysates from PC12 cells expressing calsenilin were preloaded with GDP or GTPγS, after which the proteins were immunoprecipitated with an anti-RhoA antibody and analyzed by Western blotting using anti-calsenilin and anti-RhoA antibodies. The intensities of the bands in each panel were measured and quantified for each group, and the values are expressed as the mean ± SEM of three independent experiments (*n* = 3, * *p* < 0.05; *** *p* < 0.001); (**C**) The co-localization of calsenilin with RhoA in HEK293 cells expressing either vector (VEC) or calsenilin (Cal) was assessed by double immunofluorescence staining and confocal microscopy. VEC, pEGFP-N1 vector; Cal, GFP-tagged full-length of human calsenilin; TL, total cell lysates. Glyceraldehyde 3-phosphate dehydrogenase (GAPDH) was used as a loading control. DAPI (blue) was used to counterstain the nuclei. Scale bars, 20 μm.

Calsenilin possesses partial sequence homology with RhoA (residues 66–75) and was previously identified as a Ca^2+^-independent RhoA interacting protein by proteomic analysis [7]. To assess the interaction between calsenilin and RhoA in cultured cells, we carried out co-immunoprecipitation analysis using PC12 cells stably expressing calsenilin. As shown in Figure 1, RhoA co-immunoprecipitated with calsenilin (Figure 1A). Since RhoA switches between active GTP-bound and inactive GDP-bound states, we next investigated whether the GTP- or GDP-bound state of RhoA affects its interaction with calsenilin. Cell lysates from PC12 cells expressing either empty vector alone or calsenilin were preloaded with either GDP or GTPγS, and then the co-immunoprecipitation of RhoA with calsenilin was performed. We observed that calsenilin preferentially interacted with GTPγS-bound RhoA as opposed to GDP-bound RhoA in PC12 cells expressing calsenilin (Figure 1B). We also found that calsenilin and RhoA co-localized mainly in the perinuclear region and the cytoplasm in human embryonic kidney 293 (HEK293) cells expressing (Figure 1C). In addition, calsenilin co-localized with RhoA in the cerebral cortex and hippocampus of wild-type (WT) mouse (Figure S1). These results indicate that calsenilin readily interacts with the GTP-bound state of RhoA.

### 2.2. Calsenilin Regulates RhoA Activity and the RhoA-ROCK-LIMK-Cofilin Pathway by Facilitating an Interaction between RhoA and p190RhoGAP

**Figure 2:**
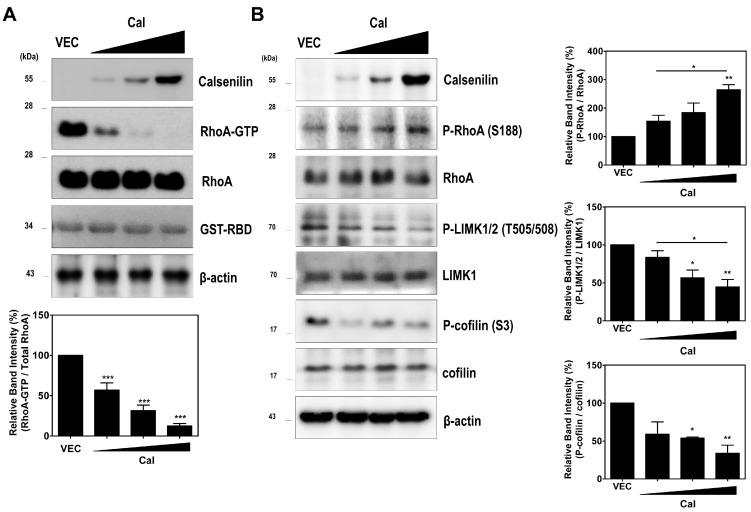
Calsenilin regulates RhoA inactivation and the RhoA-ROCK-LIMK-cofilin pathway. (**A**) PC12 cells were transiently transfected with calsenilin in a dose-dependent manner. After 24 h, the cells were lysed, and RhoA-GTP was detected by GST-Rhotekin-RBD pull-down assay; (**B**) Phosphorylation levels of RhoA, LIMK1/2, and cofilin in PC12 cells expressing calsenilin were analyzed by Western blotting. Statistical differences were determined by one-way ANOVA test with Tukey’s post hoc test (*n* = 3, * *p* < 0.05; ** *p* < 0.01; *** *p* < 0.001).

**Figure 3:**
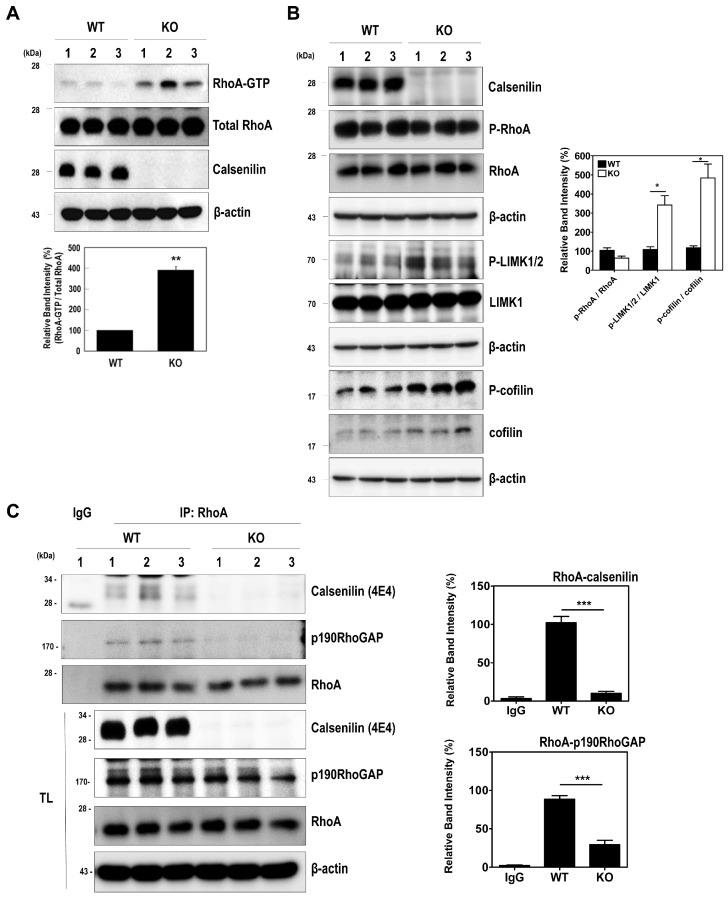
Calsenilin regulates RhoA inactivation and the RhoA-ROCK-LIMK-cofilin pathway in the mouse brain:(**A**) detection of RhoA-GTP levels in the brain of wild-type (WT) and calsenilin knockout (KO) mice; (**B**) phosphorylation levels of RhoA, LIMK, and cofilin in the whole brain lysates of WT and calsenilin-KO mice; and (**C**) co-immunoprecipitation of RhoA with calsenilin (anti-4E4) [19] and p190RhoGAP in the whole brain lysates of WT and calsenilin-KO mice. The intensities of the bands in each panel were measured and quantified for each group, and the values are expressed as the mean SEM of three independent experiments (*n* = 3, * *p* < 0.05; *** *p* < 0.001).

We next determined the effect of calsenilin on RhoA activity and the RhoA-ROCK-LIMK-cofilin pathway. As shown in Figure 2, calsenilin overexpression decreased RhoA-GTP levels in PC12 cells (Figure 2A). Moreover, calsenilin overexpression induced the phosphorylation of RhoA at Ser188 (P-RhoA), a change accompanied by dose-dependent reductions in the phosphorylation of LIMK1/2 at Thr508 and Thr505 (P-LIMK1/2) and the phosphorylation of cofilin at Ser3 (P-cofilin) (Figure 2B). To confirm these results, we examined the effect of calsenilin on RhoA activity and the phosphorylation levels of RhoA downstream effector proteins in the brains of wild-type (WT) and calsenilin-knockout (KO) mice. As expected, we observed an increase in RhoA-GTP levels (Figure 3A), as well as a decrease in P-RhoA levels and increases in P-LIMK1/2 and P-cofilin levels (Figure 3B), in the brains of calsenilin-KO mice compared to those of WT mice.

The activity of RhoA protein is tightly controlled by the following three classes of regulatory proteins: RhoGDIs, RhoGEFs, and RhoGAPs [16]. In particular, p190RhoGAP plays a pivotal regulator role in regulating RhoA activity in neuronal differentiation by interacting with the protein [17,18]. To determine a physiological interaction of calsenilin and RhoA in vivo system and examine whether calsenilin has an effect on the interaction between RhoA and p190RhoGAP, we carried out a co-immunoprecipitation assay using the brains of WT and calsenilin-KO mice. As shown in Figure 3C, calsenilin physiologically interacted with RhoA in the brains of WT mice. In addition, the capacity with which RhoA binds p190RhoGAP was significantly reduced in the brains of calsenilin-KO mice but not in the brains of WT mice. Taken together, these findings suggest that calsenilin expression efficiently inactivates RhoA, thereby affecting its downstream effector proteins, namely, LIMK and cofilin, and that calsenilin facilitates the interaction between RhoA and p190RhoGAP.

### 2.3. RhoA Preferentially Interacts with Calsenilin-CTF and Regulates RhoA Activity and the RhoA-ROCK-LIMK-Cofilin Pathway

**Figure 4:**
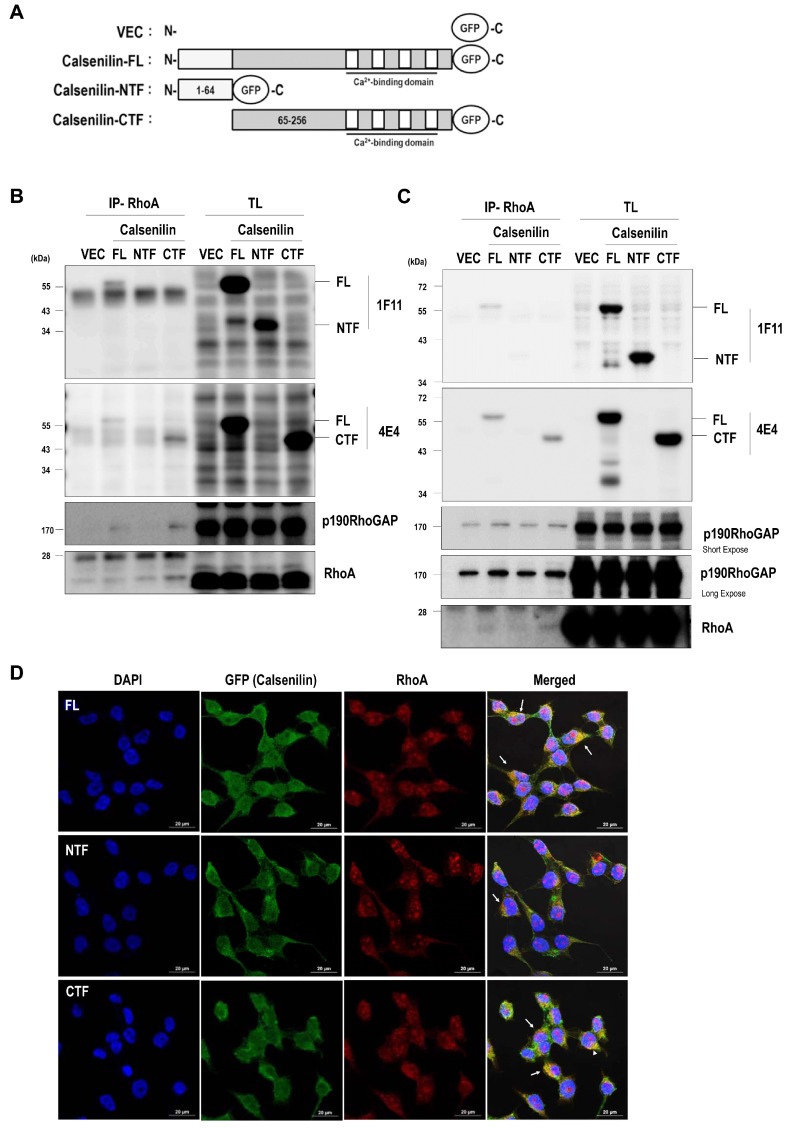
Calsenilin-CTF is responsible for the interaction with RhoA: (**A**) schematic diagram depicting the construction of the pEGFP-N1 vector, calsenilin-FL (aa 1–256), -NTF (aa 1–64), and -CTF (aa 65–256); (**B**,**C**) total lysates from HEK293 and PC12 cells expressing pEGFP-N1 vector alone (VEC), full-length calsenilin (FL), or truncated fragments (NTF or CTF) were immunoprecipitated with an anti-RhoA antibody and then analyzed by Western blotting with anti-calsenilin (1F11, amino acid 25-33; 4E4), anti-p190RhoGAP and anti-RhoA antibodies; and (**D**) the co-localization of calsenilin with RhoA in HEK293 cells expressing VEC, calsenilin-FL, -NTF, or -CTF was determined by double immunofluorescence staining using confocal microscopy. Green, calsenilin; Red, RhoA; Blue, DAPI. Scale bars, 20 μm.

To determine which domain of calsenilin interacts with RhoA, we established HEK293 and PC12 cells stably expressing pEGFP-N1 vector alone (VEC), pEGFP-N1-calsenilin-FL (residues 1–256), pEGFP-N1-calsenilin-NTF (residues 1–64), or pEGFP-N1-calsenilin-CTF (residues 65–256) (Figure 4A). We carried out a co-immunoprecipitation assay using these cells stably expressing either vector or calsenilin (FL, NTF, or CTF). As shown in Figure 4, calsenilin-FL and -CTF but not -NTF were co-immunoprecipitated with RhoA (Figure 4B,C and Figure S2). In addition, p190RhoGAP was co-immunoprecipitated with RhoA in cells stably expressing calsenilin-FL and -CTF. Using immunofluorescence staining, we observed that calsenilin-FL and -CTF co-localized with RhoA in the perinuclear region and the cytoplasm (Figure 4D). These results indicate that calsenilin-CTF is responsible for the interactions between RhoA and its regulatory proteins.

**Figure 5:**
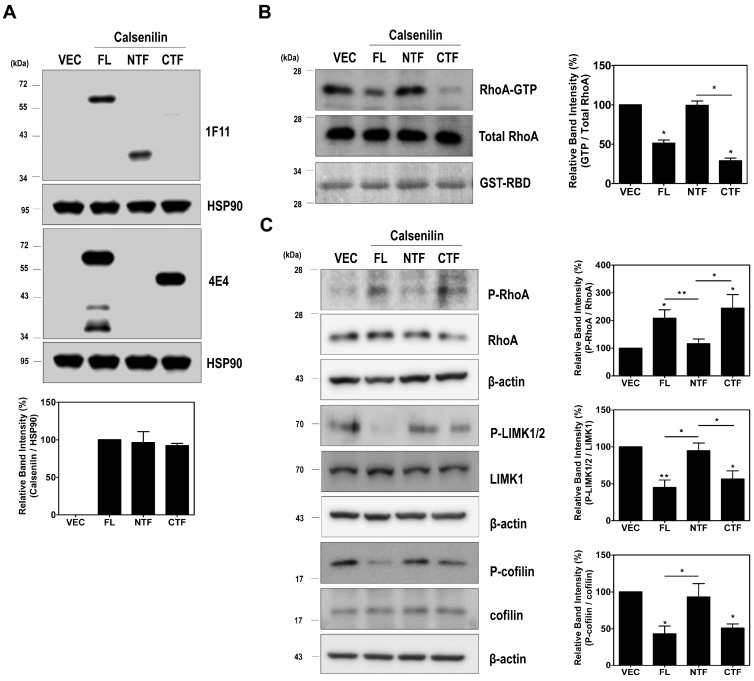
Calsenilin-CTF inactivates RhoA. (**A**) The expression level of calsenilin was determined by Western blotting with anti-1F11 and anti-4E4 antibodies; (**B**,**C**) RhoA-GTP levels and phosphorylation of RhoA and RhoA-mediated signaling proteins in PC12 cells expressing VEC, calsenilin-FL, or truncated fragments (NTF or CTF) were detected and analyzed by Western blotting. The intensities of the bands in each panel were measured and quantified for each group, and the values are expressed as the mean ± SEM of three independent experiments. Statistical differences were determined by one-way ANOVA test with Tukey’s post hoc test (*n* = 3, * *p* < 0.05; ** *p* < 0.01).

To determine whether calsenilin-CTF is also responsible for regulating RhoA activity, we performed glutathione S-transferase (GST)-Rhotekin-Rho-binding domain (RBD) pull-down assay in PC12 cells expressing either VEC or calsenilin (FL, NTF, or CTF) (Figure 5A). As shown in Figure 5B, the levels of RhoA-GTP in PC12 cells expressing calsenilin-FL and -CTF were significantly lower than those in cells expressing VEC or calsenilin-NTF. Since calsenilin-CTF is responsible for the interaction with RhoA (Figure 4B,C and Figure S2) and the induction of RhoA inactivation (Figure 5B), we examined whether calsenilin-CTF regulates the RhoA-ROCK-LIMK-cofilin pathway. As expected, calsenilin-FL and -CTF increased P-RhoA levels but decreased P-LIMK1/2 and P-cofilin levels (Figure 5C). These data suggest that the expression of calsenilin-FL and -CTF resulted in the inactivation of RhoA and, consequently, the inhibition of its downstream effector proteins including LIMK and cofilin and that the domain of calsenilin responsible for these activities is located in the CTF.

### 2.4. Calsenilin Regulates F-actin Formation

**Figure 6:**
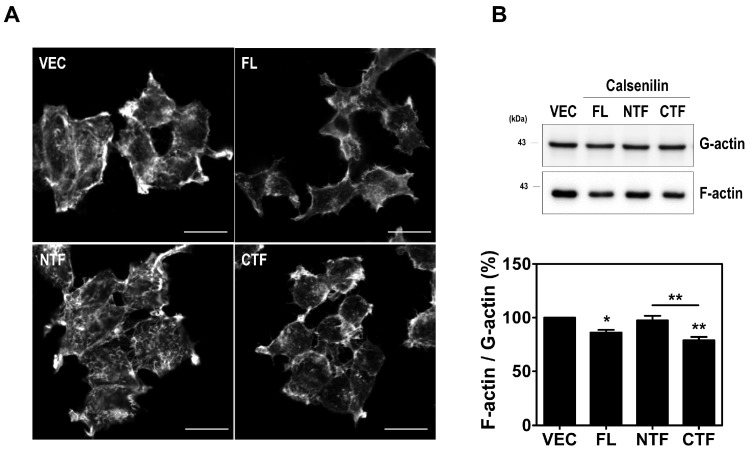
Overexpression of calsenilin-FL and -CTF decreased F-actin formation. (**A**) Immunocytochemical staining for F-actin in HEK293 cells expressing VEC, calsenilin-FL, -NTF, or -CTF. The cells were fixed with 4% PFA and permeabilized with 0.2% Triton X-100 in phosphate-buffered saline (PBS). F-actin (green) was stained with Alexa Fluor 555-phalloidin. All pictures are representative of multiple images from three independent experiments. Scale bars, 20 μm. (**B**) The expression of F-actin/G-actin as assessed by F-actin/G-actin in vivo assay in HEK293 cells expressing VEC, calsenilin-FL, -NTF, or -CTF. The intensities of the bands in each panel were measured and quantified for each group, and the values are expressed as the mean ± SEM of three independent experiments. Statistical differences were determined by one-way ANOVA test with Tukey’s post hoc test (*n* = 3, * *p* < 0.05; ** *p* < 0.01).

Previous studies have reported that RhoA acts as a pivotal regulator of actin cytoskeleton rearrangements by facilitating the formation of actin stress fibers and focal adhesions [10,20]. Thus, we investigated the effect of calsenilin on the formation of actin stress fibers in HEK293 cells and hippocampal neuronal cells derived from calsenilin-KO mice [21] expressing VEC, calsenilin-FL, -NTF, or -CTF. Stress fibers can be observed via fluorescent staining (Alexa Fluor 555-conjugated phalloidin) of F-actin. As shown in Figure 6A and Figure S3, F-actin formation was more detectable in cells expressing vector or calsenilin-NTF than in cells expressing calsenilin-FL or -CTF. To confirm this finding, we determined the changes in F-actin and G-actin levels in HEK293 cells expressing vector alone, calsenilin-FL, -NTF, or -CTF using F-actin/G-actin in vivo assay. Consistent with the above results, calsenilin-FL and -CTF expression led to a decrease in F-actin/G-actin levels compared to VEC and calsenilin-NTF expression (Figure 6B). To determine whether the contribution of calsenilin in RhoA activity mediated F-actin distribution and cell adhesion in neuronal differentiation, we performed a quantification of the fluorescence intensity profiles (F-actin) and measured the cell size (cell diameter and area) in PC12 cells expressing VEC or calsenilin-FL after nerve growth factor (NGF) treatment. As shown in Figure S4A,B, we found that cells expressing calsenilin significantly increased a fluorescence intensity at the edges of the cell in the early phase of neurite outgrowth and the size of cells in response to NGF compared to cells expressing VEC alone. Furthermore, we confirmed the F-actin-mediated cell adhesion using Cell Counting Kit-8, which is a quantitative method of evaluating cell attachment. In the cell adhesion assay, F-actin-mediated cell adhesion was significantly increased in the cells expressing calsenilin-FL or -CTF as compared to those expressing vector or calsenilin-NTF (Figure S4C,D). These findings indicate that calsenilin is involved in regulating F-actin formation and cell adhesion.

### 2.5. Calsenilin-CTF Induces Neurite Outgrowth through RhoA-Mediated Signaling

**Figure 7:**
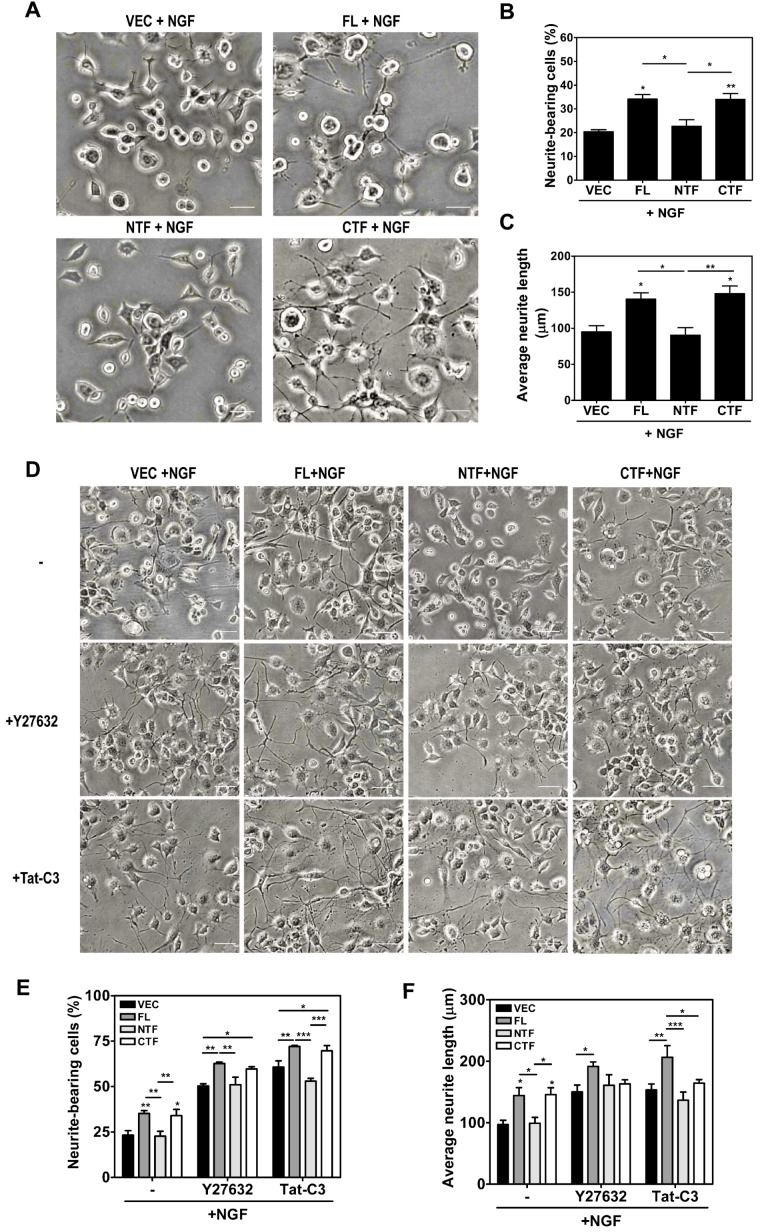
Overexpression of calsenilin FL and CTF enhanced neurite outgrowth by decreasing RhoA activity and RhoA-mediated signaling. (**A**–**C**) Morphological changes in PC12 cells expressing VEC, calsenilin FL, -NTF, or -CTF treated with 50 ng/mL NGF 2.5S for 72 h. All values are expressed as the mean ± SEM of three independent experiments. Statistical differences were determined by one-way ANOVA test with Tukey’s post hoc test (*n* = 10–20 cells per treatment group, * p < 0.05; ** *p* < 0.01); (**D**–**F**) The cells were incubated with or without 10 μM Y27632 and 1 μg Tat-C3 for 72 h after NGF treatment. The number of neurites per cell was determined by counting all the processes longer than two cell diameters in length. The changes in neurite outgrowth were measured by using INFINITY analysis software. The data represent the mean ± SEM of three independent experiments. Statistical data were obtained by two-way ANOVA with Bonferroni’s post hoc test (*n* = 10–20 cells per treatment group, * *p* < 0.05; ** *p* < 0.01; *** *p* < 0.001). Scale bars, 100 μm.

It is known that RhoA inactivation induces neurite outgrowth in PC12 cells [22]. Therefore, we investigated whether the expression of calsenilin, specifically calsenilin-CTF, promotes neurite outgrowth in PC12 cells subjected to NGF treatment and found that the expression of calsenilin-FL or -CTF was associated with increases in the number of neurite-positive cells and neurite length in response to NGF compared to VEC or calsenilin-NTF (Figure 7A–C). To elucidate whether the increases in neurite outgrowth regulated by calsenilin is due to RhoA-mediated signaling, cells were pretreated with Tat-C3 (a specific inhibitor of RhoA, ADP-ribosylation at Asn41) and Y27632 (an inhibitor of ROCK). Interestingly, Tat-C3 and Y27632 treatment significantly increased the percentage of neurite-bearing cells, neurite length, and the number of neurites in cells (Figure 7D–F and Table S1). These data indicate that calsenilin-CTF is responsible for the neurite outgrowth and cellular signal transduction related to RhoA inactivation.

## 3. Discussion

Calsenilin is a member of the neuronal Ca^2+^-binding protein family that has been shown to modulate disease pathogenesis by regulating Aβ42 production, neuronal cell death, pain, synaptic depression, and learning and memory in a Ca^2+^-dependent manner [6,23,24,25]. A recent study demonstrated that calsenilin can directly interact with RhoA in a Ca^2+^-independent manner using Liquid Chromatography/Mass Spectrometry (LC/MS) analysis [7], and we found that calsenilin shared a partial sequence homology with RhoA. However, the mechanism responsible for the direct regulation of RhoA activity and its related effector proteins through calsenilin is still unclear.

**Figure 8:**
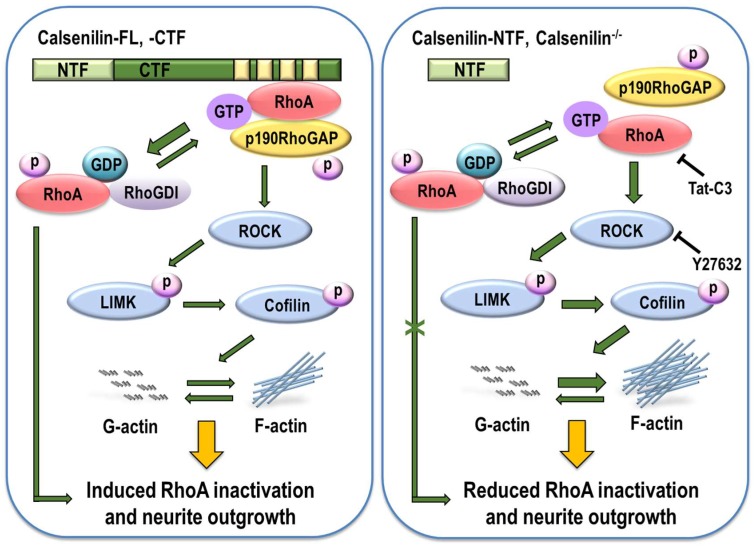
Overexpression of calsenilin-FL and -CTF enhanced RhoA inactivation and neurite outgrowth. In calsenilin-FL and -CTF expressing cells, calsenilin increased the phosphorylation of RhoA, enhancing the interaction between RhoA and p190RhoGAP. This complex led to the inactivation of RhoA and its downstream effector proteins. Subsequently, RhoA inactivation decreased actin polymerization and increased neurite outgrowth. In contrast, depletion of calsenilin prevented RhoA inactivation and neurite outgrowth by interfering with the interaction.

In this study, we demonstrate that calsenilin has a novel functional role in regulating RhoA activity and the RhoA-ROCK-LIMK-cofilin pathway by interacting with RhoA and p190RhoGAP in in vitro and in vivo systems (Figure 8), in concert with increased phosphorylation of RhoA and decreased phosphorylation of downstream effector proteins (i.e., LIMK and cofilin). In addition, previous reports have demonstrated that interaction of RhoA and p190RhoGAP regulates neurite outgrowth in PC12 cells through RhoA inactivation [17,18,22,26,27,28]. We also demonstrated that calsenilin depletion inhibited neurite outgrowth and impaired actin cytoskeleton organization by a reduced interaction of RhoA with p190RhoGAP, a well-known molecule that acts as an effector protein for cell invasion and migration by promoting membrane protrusion and polarity [29,30].

The RhoA activity is involved in neuronal development and differentiation by regulating RhoGAPs, the RhoA-ROCK-LIMK-cofilin pathway, and PI3K/Akt signaling [13,17,18,22,31,32,33]. Following the expression of calsenilin in a dose-dependent manner, the level of RhoA-GTP was significantly decreased with a reduction in phosphorylation of LIMK1/2 and cofilin (an inactive form) without changes in the total levels of RhoA, LIMK1, or cofilin (Figure 2B). Furthermore, we demonstrated that depletion of calsenilin increased the levels of RhoA-GTP as a result of a decrease in the interaction between RhoA and p190RhoGAP (Figure 3). These findings suggest that calsenilin plays an important role in the regulation of RhoA activity via an interaction with RhoA and p190RhoGAP. In addition, inhibiting RhoA and ROCK via overexpression of a dominant-negative mutant RhoA (N19) or treatment with Tat-C3 and Y27632 enhanced neurite outgrowth in PC12 cells exposed to NGF, basic fibroblast growth factor (bFGF), and cAMP [22,34], suggesting that calsenilin exerts its influence on neuronal differentiation through the modulation of RhoA activity and ROCK.

Previous reports demonstrate that the effects of Ca^2+^ on neurite outgrowth are related to the regulation of cytoskeletal rearrangement in various neuronal cell lines [35], and that excessive decreases or increases in intracellular Ca^2+^ levels may inhibit neurite outgrowth [36,37]. Although we did not test RhoA activity or the interaction between calsenilin and RhoA in the presence or absence of Ca^2+^, our data showed that calsenilin-CTF, which contains 4 Ca^2+^-binding EF-hands domain [4], is primarily responsible for the interaction between RhoA and p190RhoGAP, an event that coincides with decreases in RhoA activity. It has been reported that RhoA activation significantly increased intracellular Ca^2+^ levels, and that the inhibition of the increase in Ca^2+^ levels enhanced neurite outgrowth [35]. Therefore, Ca^2+^ may be involved in the calsenilin-mediated RhoA signaling pathway and neurite outgrowth.

Rho GTPases participate in various functions, including actin dynamics, cell migration, proliferation, differentiation, transcriptional regulation, cell cycle progression, and membrane trafficking [8,9,10]. RhoA, Cdc42, and Rac1 are the most extensively characterized Rho GTPases and are key effectors in the regulation of the microtubules and the actin cytoskeleton in neuronal development [20,28,38]. Various proteins related to the RhoA-Cdc42-Rac1 pathways, including Wiskott-Aldrich Syndrome protein (WASP), p21-activated kinase (PAK), Cyclin-dependent kinase 5 (CDK5), Rho-kinase and Diaphanous homolog 1 (Dia1), have been implicated in microtubule and actin dynamics. In addition, Rac1 and Cdc42 are involved in growth cone lamellipodia formation, which is essential for neurite outgrowth, and Rac1 and Cdc42 activation and RhoA activity inhibition enhance neurite outgrowth by post-translational mechanisms; both pathways functionally connect with ROCK [39,40]. We also found that overexpression of calsenilin induced Rac1 and Cdc42 activation (Figure S5) and increased neurite outgrowth. Therefore, the possibility that calsenilin promotes neurite outgrowth from PC12 cells through the activation of Cdc42 and Rac1, which is induced by RhoA inactivation, cannot be excluded. It is noteworthy that RhoA antagonizes Rac1 [41,42], RhoA/ROCK phosphorylates FilGAP, a GAP for Rac1, leading to Rac1 inactivation [43], while activated Rac1 stimulates the activity of p190RhoGAP by promoting its phosphorylation, leading to RhoA inactivation [44,45].

To demonstrate the interaction between calsenilin and RhoA, we used several types of cell lines as in vitro experimental models. In particular, PC12 cells are a widely used in vitro model for neuronal differentiation after NGF treatment [46,47]. It is known that NGF-differentiated PC12 cells extend long neuron-like processes (neurites), which are connected to each other by synapse-like structures [48] with swellings that accumulate small clear vesicles and dense core vesicles [49,50,51]. In addition, previous studies have demonstrated that calsenilin is associated with trafficking of synaptic vesicles and may regulate neurotransmitter release [52]. Future studies are needed to understand the physiological role of calsenilin in the regulation of synaptogenesis and synaptic plasticity.

Taken together, our results demonstrate that calsenilin has a physiological role in neuronal differentiation that contributes to RhoA inactivation, leading to neuronal differentiation. These findings are important for understanding the mechanisms of calsenilin-regulated neuronal differentiation and its mediated signaling pathway.

## 4. Materials and Methods

### 4.1. Cell Culture, Transfection, and Generation of Stable Cell Lines

PC12 cells, which are derived from a pheochromocytoma of the rat adrenal medulla [48], were grown in Roswell Park Memorial Institute (RPMI) 1640 medium (HyClone, South Logan, UT, USA) containing 10% horse serum (HS; Thermo Fisher Scientific, Rockford, IL, USA), 5% fetal bovine serum (FBS; HyClone), and 1% penicillin/streptomycin (P/S; HyClone) at 37 °C under 5% CO_2_. Hippocampal neuronal cells derived from calsenilin-KO mice [53] were newly established by transfection with an SV40 large T-antigen-containing vector (pSV3neo; Invitrogen, Carlsbad, CA, USA) as described previously [21,54]. The hippocampal neuronal cells, as well as HEK293 cells, were cultured in Dulbecco’s Modified Eagle’s Medium (DMEM; HyClone) containing 10% FBS and 1% P/S. Transient transfections were carried out with Lipofectamine 2000 according to the manufacturer’s instructions (Thermo Fisher Scientific). Cells stably expressing a pEGFP-N1 neomycin resistance vector (VEC), GFP-tagged human calsenilin-FL, calsenilin-NTF, or calsenilin-CTF were generated and maintained in the presence of 100 μg/mL Geneticin (G418; Thermo Fisher Scientific).

### 4.2. Construction of Recombinant Plasmids

The cDNAs encoding the FL, NTF or CTF of human calsenilin in pcDNA3.1/Zeo^(+)^ (Invitrogen) have been described previously [4]. The calsenilin–EGFP constructs were made by inserting the FL, NTF, or CTF of calsenilin into the pEGFP-N1 vector (BD Biosciences, Mississauga, ON, Canada) after amplifying the sequences from an existing pcDNA3.1/Zeo^(+)^ calsenilin with the following primers (the endonuclease sites are underlined): FL-EGFP, forward 5′-CACTCGAGGCCACCATGCAGCCGGCTAAGGAA-3′ (XhoI) and reverse 5′-GAGAAGCTTGATGACATTCTCAAACTG-3′ (HindIII); NTF-EGFP, forward 5′-CACTCGAGGCCACCATGCAGCCGGCTAAGGAA-3′ (XhoI) and reverse 5′-GAGAAGCTTGTCGCTGCTATCTGAGCC-3′ (HindIII); and CTF-EGFP, forward 5′-CACTCGAGGCCACCATGAGTGAGCTGGAGCTG-3′ (XhoI) and reverse 5′-GAGAAGCTTGATGACATTCTCAAACTG-3′ (HindIII). For the plasmid amplification, DH5α competent cells (Invitrogen) were transformed with the recombinant plasmids and grown in Luria-Bertani (LB) media. The bacterial cells were harvested and plasmid purification was performed with a Qiagen Plasmid Midi Kit (Qiagen, Valencia, CA, USA), according to the manufacturer’s instructions. The sequences of all of the constructs were confirmed by DNA sequencing (Cosmo Genetech, Seoul, Korea).

### 4.3. Animals

Wild-type (WT) and calsenilin-KO mice were generated as previously described [53] and housed in a clean facility under natural light-dark cycle conditions (12 h/12 h light/dark cycle) and examined at 8–10 weeks of age. For Western blot analysis and co-immunoprecipitation assay, the brain tissues of 3-month-old WT and calsenilin-KO mice were collected and stored at −70 °C until use. All experiments were performed in accordance with Korean laws and with the approval of the Hallym Medical Center Institutional Animal Care and Use Committee (HMC2017-0-0102-1; 28 February 2017).

### 4.4. Western Blot Analysis

Cells were washed with ice-cold phosphate-buffered saline (PBS) and lysed with a modified RIPA buffer containing 50 mM Tris-HCl pH 7.4, 150 mM NaCl, 1% Triton X-100, 0.1% sodium dodecyl sulfate, 0.5% sodium deoxycholate, 1 mM ethylenediaminetetraacetic acid (EDTA), protease inhibitors (Pierce Biotechnology, Rockford, IL, USA), 1 mM Na_3_VO_4_, and 1 mM NaF. The cell lysates were centrifuged at 15,000× *g* for 15 min at 4 °C, and the protein concentrations in the supernatants were analyzed using a BCA Protein Assay Kit (Thermo Fisher Scientific). Equal amounts of proteins (40 µg/lane) were separated using sodium dodecyl sulfate-polyacrylamide electrophoresis, transferred onto 0.45-µm pore of polyvinylidene fluoride (PVDF) membranes (Merck Millipore, Lake Placid, NY, USA) and blocked with 5% skim milk in 1× PBS containing 0.1% Tween 20 (PBST) for 1 h at room temperature (RT). The following primary antibodies were added to the membranes, which were incubated overnight at 4 °C: anti-RhoA, anti-cofilin (SantaCruz, Dallas, TX, USA), anti-phospho-RhoA, anti-phospho-LIMK1/2, anti-LIMK1, anti-GAPDH (Abcam, Cambridge, MA, USA), anti-p190RhoGAP (Merck Millipore), anti-phospho-cofilin (Cell Signaling Technology, Danvers, MA, USA), and anti-β-actin (Sigma-Aldrich, St. Louis, MO, USA). The membranes were washed with PBST 3 times for 10 min each and then incubated with the following secondary antibodies for 1 h: goat anti-mouse IgG (Thermo Fisher Scientific, Waltham, MA, USA) or goat anti-rabbit IgG (Thermo Fisher Scientific) conjugated with horseradish peroxidase (HRP). The membranes were then washed with PBST 3 times for 10 min each, and the membrane-bound antibodies were detected by chemiluminescence and captured images using the ImageQuantTM LAS4000 apparatus (GE Healthcare Life Science, Piscataway, NJ, USA).

### 4.5. Co-Immunoprecipitation

For the immunoprecipitation experiments, the cells and brain tissues were lysed with modified RIPA buffer containing 50 mM Tris-HCl pH 7.4, 150 mM NaCl, 1% NP-40, 0.25% sodium deoxycholate, 1 mM EDTA, and protease inhibitors (Pierce Biotechnology). Total lysates from the cultured cells and brains were centrifuged at 15,000× *g* for 15 min, and then the supernatants were precleared with Protein A-conjugated Sepharose 4B beads and normal IgG for 2 h at 4 °C. The supernatants were then incubated with new beads and the appropriate primary antibodies 2 h at 4 °C. After incubation, the supernatants were centrifuged at 3000× *g* for 15 s and the beads were washed with lysis buffer 3 times for 10 min. The beads were boiled with 2× Laemmli sample buffer containing 2-mercaptoethanol for 15 min at 95 °C. The samples were electrophoresed and then analyzed by Western blot with the appropriate antibodies.

### 4.6. In Vitro Loading of GDP and GTPγS onto GTP-Binding Proteins

Cell lysates (500 μg/mL proteins in 500 μL) were incubated with 10 mM EDTA (pH 8.0). Next, 0.1 mM GTPγS or 1 mM GDP was added to the cell lysates, which were incubated under constant agitation for 15 min at 30 °C. The reaction was terminated by the addition of MgCl_2_ at a final concentration of 60 mM on ice.

### 4.7. Glutathione-S-Transferase (GST)-Rhotekin-RBD Pull-Down Assay for RhoA Activation

The cells were harvested and washed with PBS, and then they were lysed in binding/washing/lysis buffer containing 25 mM Tris-HCl (pH 7.4), 150 mM NaCl, 5 mM MgCl_2_, 1% NP-40, 1 mM DTT, 5% glycerol, 1 mM EDTA, 1 mM ethylene glycol-bis(2-aminoethylether)-*N,N,N′,N′*-tetraacetic acid with protease inhibitors (Pierce Biotechnology), 10 mM NaF, and 1 mM Na_3_VO_4_. The lysates were centrifuged at 13,000× *g* for 10 min at 4 °C, after which the supernatant was incubated with GST-Rhotekin-RBD to detect RhoA-GTP. The beads were washed with binding/washing/lysis buffer 3 times. The bound proteins were eluted with 2× Laemmli sample buffer by boiling, and the samples were electrophoresed and analyzed by Western blotting with an anti-RhoA antibody.

### 4.8. Immunofluorescence Staining

Cells (5 × 10^4^ cells/35-mm dish) were exposed to 50 ng/mL of NGF for 72 h, rinsed with 1X PBS 3 times for 10 min each and then fixed with 4% paraformaldehyde (PFA) for 10 min. The fixed cells were rinsed with 1× PBS 3 times for 10 min each and then permeabilized with 1× PBS containing 0.1% Triton-X100 for 10 min at RT. The cells were then blocked with 1× PBS containing 1% BSA and 5% goat serum for 1 h at RT after being rinsed with 1× PBS 3 times for 10 min each. After blocking, the cells were incubated with the following primary antibodies in 1% BSA and 5% goat serum in 1× PBS overnight at 4 °C: anti-calsenilin (anti-1F11 or anti-4E4) [21] and anti-RhoA (SantaCruz). The cells were subsequently incubated with either Alexa Fluor 568 goat anti-rabbit IgG or Alexa Fluor 488 goat anti-mouse IgG antibodies (Invitrogen) for 1 h, and then they were incubated with Alexa 488 and Alexa 568 fluorescent secondary antibodies (Thermo Fisher Scientific) for 1 h. After being rinsed with PBS, the cells were mounted in 4′,6-diamidino-2-phenylindole (DAPI)-containing Vectashield Mounting Medium (Vector Laboratories, Burlingame, CA, USA) to label the nuclei and visualized using a confocal laser scanning microscope (LSM 700; Carl Zeiss, Oberkochen, Germany).

### 4.9. Quantitation of F-Actin and G-Actin

The amounts of filamentous actin (F-actin) and globular actin (G-actin) in cultured cells were determined using an G-actin/F-actin in vivo assay kit (Cytoskeleton, Denver, CO, USA), according to the manufacturer’s instructions.

### 4.10. Adhesion Assays

Cells were suspended RPMI 1640 medium containing 10% HS, 5% FBS, and 1% P/S and plated on poly-d-lysine (Sigma-Aldrich) precoated 48-well plates at a density of 5 × 10^4^ cells/well. After 1 h, the cells were washed with 1× PBS 3 times, and the remaining suspended cells were removed. The adherent cells were quantified using Cell Counting Kit-8 (Dojindo Laboratories, Kumamoto, Japan) at 450 nm wavelength absorbance in a VersaMax Microplate Reader (Molecular Devices, Sunnyvale, CA, USA).

### 4.11. Neurite Outgrowth Analysis

PC12 cells were plated on 35-mm culture dishes coated with poly-d-lysine solution at a density of 5 × 10^4^ cells/well. After 12 h, the cells were incubated with DMEM containing with 1% HS, 0.5% FBS, 1% P/S, and 50 ng/mL NGF (Merck Millipore) for 72 h. The percentage of cells bearing neurites at least two cell bodies in length was determined by counting at least 100 single cells/3 arbitrary positions per dish [55]. Cells were visualized using a phase-contrast microscope (Nikon TS100, Nikon, Tokyo, Japan). The cell images were captured using INFINITY ANALYZE software (Release 6.5, Lumenera, Ottawa, ON, Canada), and used to measure neurite outgrowth including the percentage of neurite-bearing cells and neurite length and number.

### 4.12. Statistical Analysis

Statistical analyses were performed and graphs were generated using GraphPad Prism software (GraphPad Prism 4, GraphPad software, La Jolla, CA, USA). Statistical differences with band intensities, neurite-bearing cells, neurite lengths and cell adhesion between vector and overexpressing of calsenilin constructs or WT and calsenilin-KO mice brains were determined by one-way analysis of variance (ANOVA) followed by Tukey’s post hoc test and two-way ANOVA was used when two parameters are simultaneously compared between distinct treatments and calsenilin constructs with Bonferroni’s post hoc test to determine significant differences between each group. The data are presented as means ± SEM. Statistical significance was reached at *p* < 0.05.

## Supplementary Materials

Supplementary Materials can be found at http://www.mdpi.com/1422-0067/19/4/1196/s1.

## Abbreviations

GAP	GTPase-activating protein
NGF	nerve growth factor
ROCK	Rho-associated kinase
RhoGDI	Rho GDP-dissociation inhibitor
GST-Rhotekin-RBD	glutathione S-transferase-Rhotekin Rho-binding domain
DREAM	downstream regulatory element antagonist modulator
CREB	cAMP response element binding protein
WASP	Wiskott-Aldrich Syndrome protein
PAK	p21-activated kinase
CDK5	Cyclin-dependent kinase 5
