# Supplementary material for: Calsenilin, a Presenilin Interactor, Regulates RhoA Signaling and Neurite Outgrowth
Source: Int J Mol Sci. 2018 Apr 13;19(4):1196. doi: 10.3390/ijms19041196 (PMC5979497; doi:10.3390/ijms19041196)
Supplement: Supplementary file 1 [file ijms-19-01196-s001.pdf]

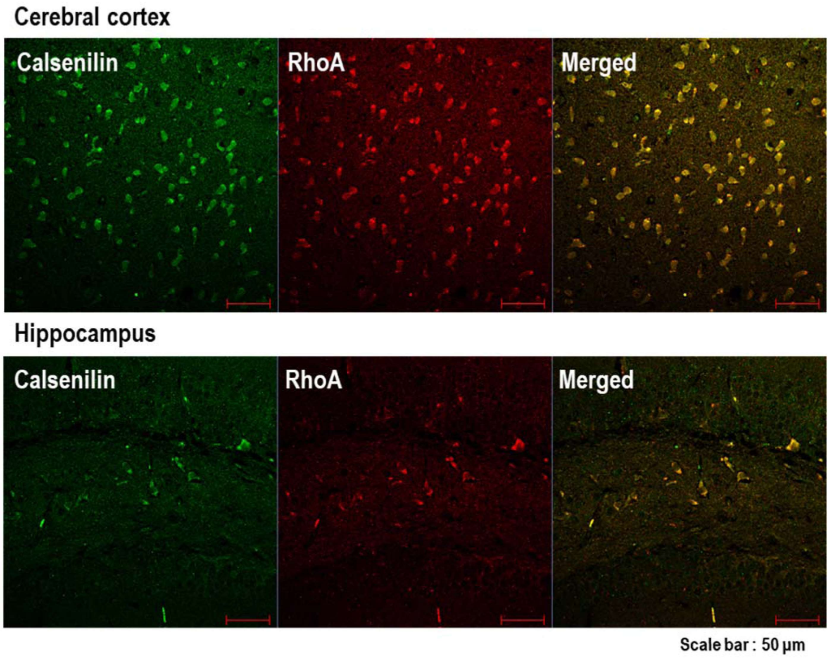

Figure S1. **Calsenilin colocalized with RhoA in the mouse brain**. Co-localization of calsenilin with RhoA in the cerebral cortex and hippocampus of wild-type (WT) mouse was assessed by double immunofluorescence staining. DAPI (blue) was used to counterstain the nuclei. Scale bars, 50 μm.

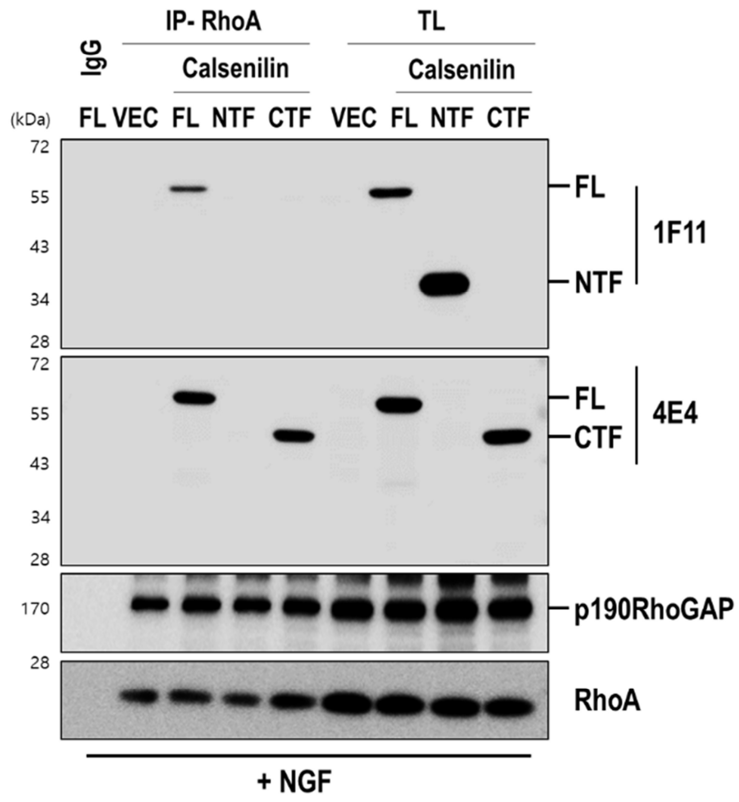

Figure S2. **Calsenilin interacts with RhoA in NGF-treated PC12 cells.** PC12 cells stably expressing pEGFP-N1 vector alone (VEC), calsenilin-FL, -NTF, or -CTF were treated with NGF for 48 h and co-immunoprecipitated with an anti-RhoA antibody followed by Western blotting with anti-calsenilin (1F11 or 4E4), anti-p190RhoGAP and anti-RhoA antibodies.

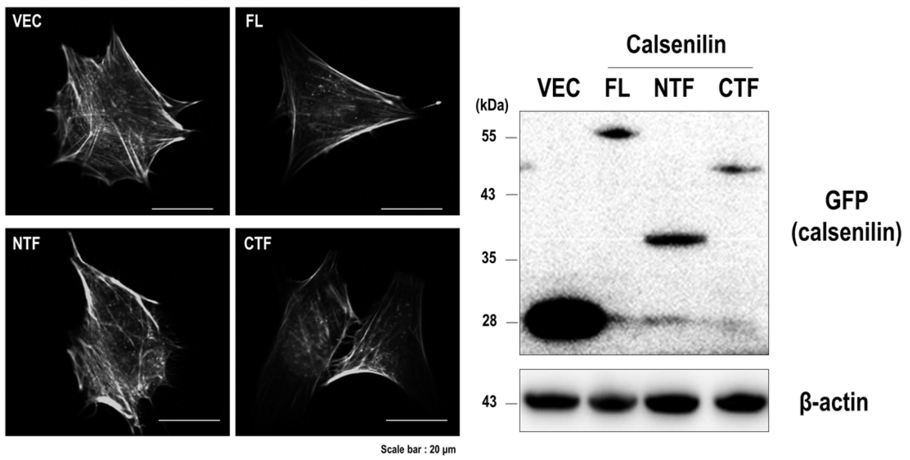

Figure S3. Overexpression of calsenilin-FL and -CTF decreased F-actin formation in hippocampal neuronal cells. Immunocytochemical staining for F-actin in hippocampal neuronal cells stably expressing VEC, calsenilin-FL, -NTF, or -CTF that were fixed with 4% PFA, and permeabilized with 0.2% Triton X-100 in PBS. F-actin (green) was stained with Alexa Fluor 555-phalloidin. All pictures are representative of multiple images from three independent experiments.Scale bars, 20 μm.

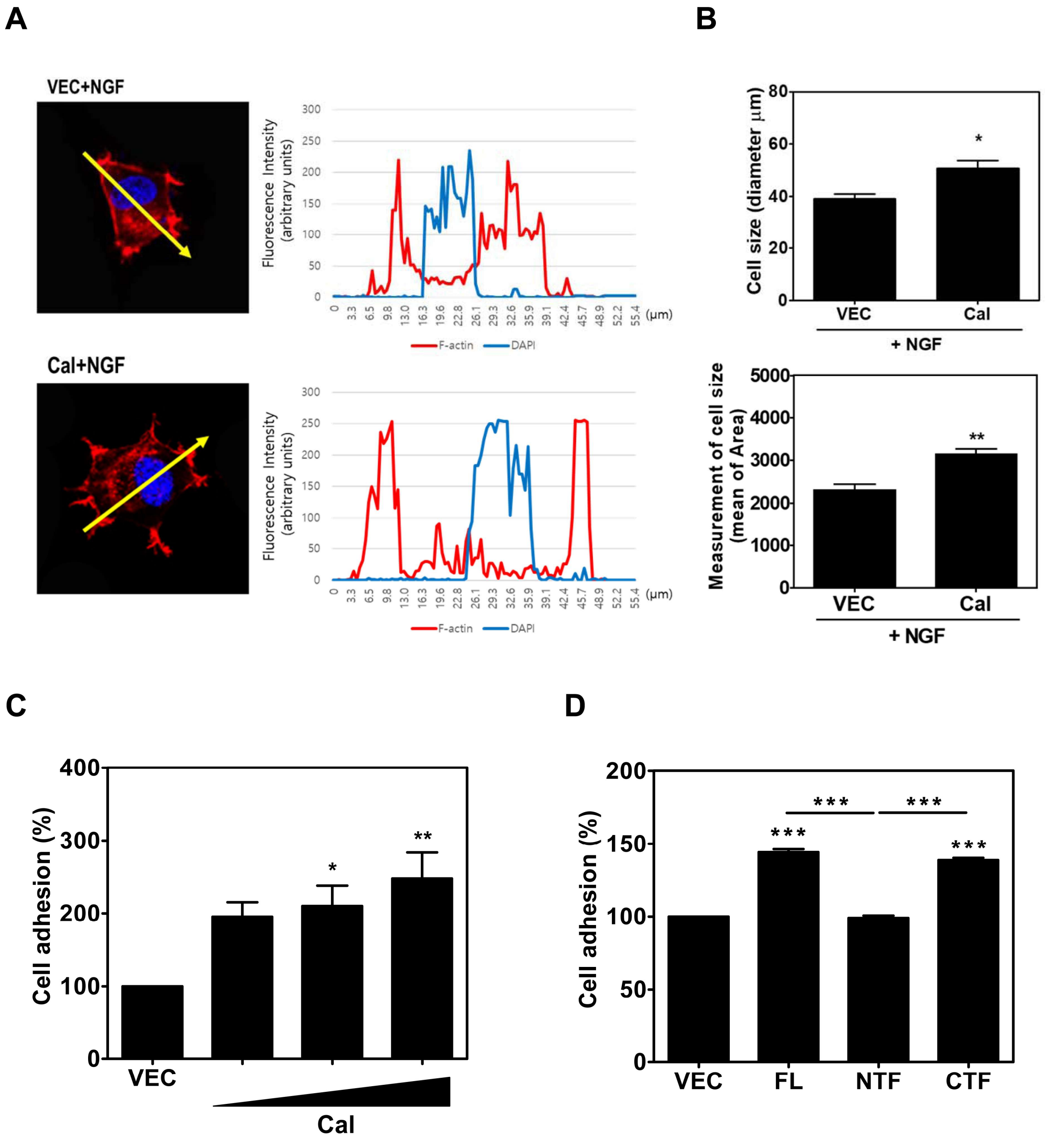

Figure S4. **Calsenilin regulates the RhoA-mediated F-actin polymerization and cell adhesion in PC12 cells**. (A) PC12 cells expressing pEGFP-N1 vector alone (VEC) and full-length calsenilin (FL) quantified the distribution of F-actin intensity and profiles across a line (yellow) in cells stimulated with NGF for 48 h using double immunofluorescence staining. DAPI (blue) was used to counterstain the nuclei. (B) Measurement of cell size were measured the diameter of cell and mean of area (cell size) using image J software. The data are representative of two independent experiments and analyzed using one-way ANOVA test with Tukey's *post hoc* test (n=10-20 cells per each group, $*p < 0.05$; $**p < 0.01$). (C) PC12 cells expressing calsenilin, (D) VEC, calsenilin-FL, -NTF, or -CTF that were seeded at a density of $5x10^4$ cells/well were allowed to attach to poly-D-lysine-coated cell culture plates for 1 h (n=4). The adherent cells were quantified an optical density (OD) of 450 nm using Cell Counting Kit-8 (a colorimetric assay). Statistical differences were determined by one-way ANOVA test with Tukey's *post hoc* test (n=4, $*p < 0.05$; $**p < 0.01$; $***p < 0.001$).

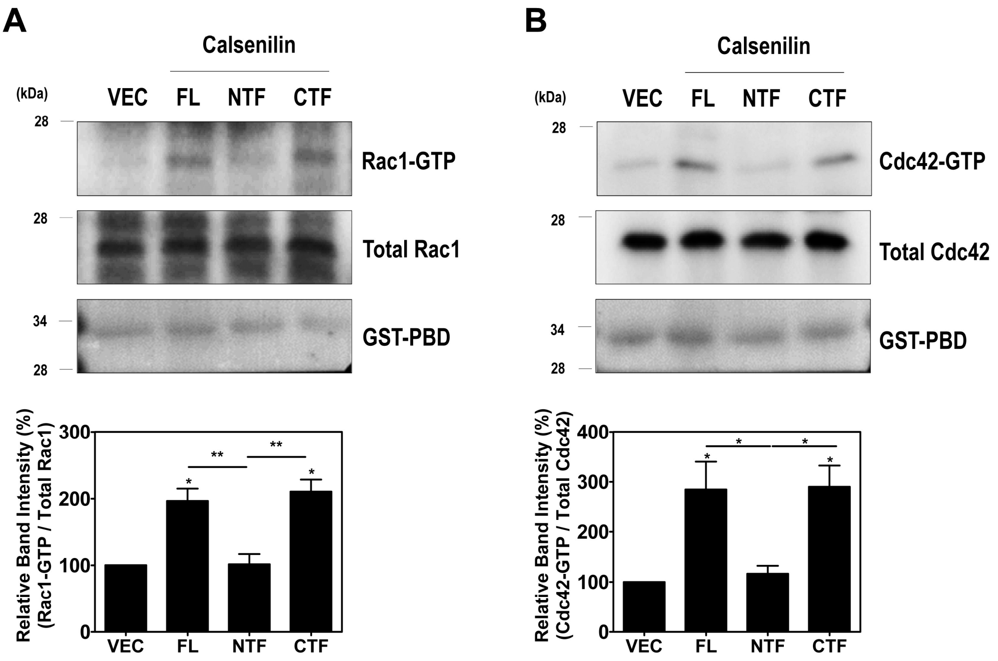

Figure S5. Calsenilin-CTF is involved in Rac1 and Cdc42 activation. (A and B) Detection of Rac1-GTP and Cdc42-GTP by GST- p21-activated kinase 1 (PAK1)-PBD pull-down assay in PC12 cells expressing VEC, calsenilin-FL, -NTF, or -CTF. The data are expressed as the mean ± SEM of three independent experiments. Statistical differences were determined by one-way ANOVA test with Tukey's *post hoc* test (n=3, *p < 0.05; **p < 0.01).

Table S1. Distribution of neurite numbers in PC12 cells expressing calsenilin under various treatments

Cell	Treatment	Percentage cells bearing varying number of neurites			
		1	2	3	≥4
VEC	NGF	38.87 (±3.93)	37.59 (±4.27)	15.85 (±3.36)	7.69 (±2.03)
	NGF+Y27632	18.34 (±4.50)	27.64 (±3.25)	28.05 (±1.78)	25.96 (±5.34)
	NGF+Tat-C3	13.36 (±2.07)	18.70 (±3.62)	26.65 (±4.10)	41.29 (±1.91)
Calsenilin-FL	NGF	23.36 (±4.24)	33.91 (±4.33)	21.47 (±4.26)	21.26 (±3.29)
	NGF+Y27632	11.53 (±7.22)	23.71 (±4.24)	25.23 (±4.32)	39.53 (±7.50)
	NGF+Tat-C3	11.74 (±3.99)	20.82 (±1.97)	21.77 (±2.85)	45.67 (±8.74)
Calsenilin-NTF	NGF	39.73 (±4.57)	36.36 (±3.53)	16.10 (±3.07)	7.81 (±1.94)
	NGF+Y27632	13.84 (±4.80)	35.97 (±2.14)	25.89 (±5.48)	24.30 (±1.83)
	NGF+Tat-C3	17.23 (±3.40)	27.23 (±3.59)	23.81 (±2.98)	31.73 (±4.56)
Calsenilin-CTF	NGF	22.89 (±5.93)	30.30 (±1.04)	21.68 (±3.14)	25.12 (±4.60)
	NGF+Y27632	9.15 (±1.44)	25.99 (±3.26)	28.37 (±1.85)	36.49 (±3.16)
	NGF+Tat-C3	5.78 (±0.98)	17.52 (±5.33)	36.35 (±6.91)	40.35 (±1.09)
